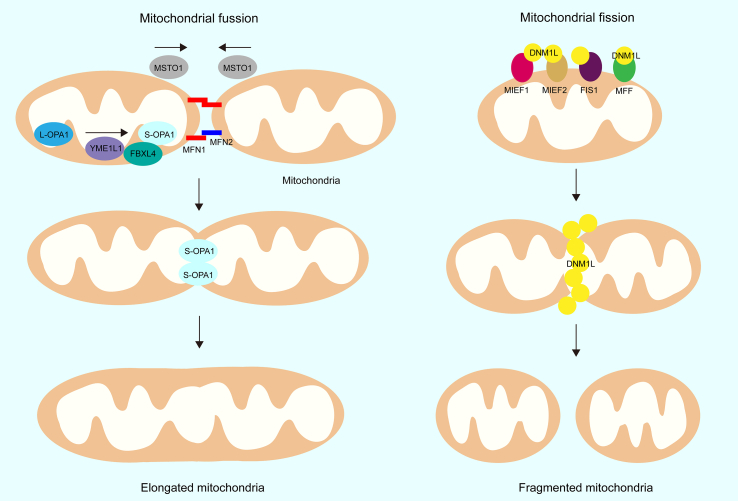# Comprehensive analysis of mitochondrial dynamic-related genes on their functions and prognostic values for glioblastoma multiforme

**DOI:** 10.1016/j.gendis.2023.101084

**Published:** 2023-09-09

**Authors:** Zhu Xie, Wei Hua, Hongyan Wang

**Affiliations:** a Shanghai Key Laboratory of Metabolic Remodeling and Health, Institute of Metabolism and Integrative Biology, Obstetrics and Gynecology Hospital, State Key Laboratory of Genetic Engineering, Fudan University, Shanghai 200433, China; b Institute of Reproduction and Development, Shanghai Institute of Planned Parenthood Research, NHC Key Laboratory of Reproduction Regulation, Children's Hospital, Fudan University, Shanghai 200433, China; c Institutes of Biomedical Sciences, Huashan Hospital, Fudan University, Shanghai 200435, China

Glioblastoma multiforme (GBM) is the most malignant intracranial tumor in adults and its unique pathology leads to limited therapeutic benefits.^1^^,^^2^ Mitochondrial fusion and fission play an important role in carcinogenesis; fragmented mitochondria promote tumor cell proliferation and prolonged mitochondria lead to tumor cell apoptosis.^3^ Therefore, profiling the function and prognostic value of mitochondrial dynamics-related genes (MDRGs) is of great interest for GBM precision treatment. Here we focused on the expression, function, and genetic alterations of MDRGs and identified new DNA methylation sites being significantly associated with the survival of GBM patients using available data in public databases. We then constructed the tumor prognostic model that accurately forecast the survival of GBM patients based on MDRGs' signature. Furthermore, it was demonstrated that the expression of MDRGs and risk factors served as independent indexes to estimate the level of immune infiltration in tumor microenvironment and response to targeted immune checkpoints in GBM patients. Notably, we filtered out acetaminophen targeting risk genes as a candidate drug for GBM treatment after clarifying risk genes' contribution to the cancer process at the single-cell level. Overall, the new biomarkers, prognostic model, and targeted drugs characterized in this study provide a novel perspective for GBM management.

To verify the mitochondrial morphology in glioma cells, we first examined the expression of MDRGs in tumor tissues. The results showed that MFN2, MFF, OPA1, and MSTO1 expression was significantly down-regulated and YME1L1 and FIS1 expression was up-regulated in tumor tissues (Fig. S1A–L, 2A–Z). Our differential expression gene analysis results supported that MDRGs contribute to the pathogenesis of gliomas. However, it remains inadequate as an indicator of mitochondrial morphology in gliomas because the expression of most genes related to mitochondrial fusion and fission decreased considerably. We then performed functional enrichment analysis to annotate the underlying mechanism and biological functions of MDRGs. GO and KEGG analysis showed that selected MDRGs were important for mitochondrial dynamics (Fig. S3C–F). Additionally, the co-expression analysis results revealed that MDRGs' expression was significantly associated with each other (Fig. S3A).

To explore the genetic alterations of MDRGs in GBM, copy number variations (CNV) and single nucleotide variants (SNV) of the above genes in the GSCA database were verified. At least one SNV in 251 samples out of 403 GBM patients was found (62.28%). *YME1L1* had the highest mutation frequency and *MFN1* showed the top missense mutation frequency (Fig. S3B). CNV alteration analysis indicated that the most frequent deletion was *YME1L1* and the amplified was *FIS1*, and both were heterozygous alterations, as presented in Table S1. DNA methylation sites display a strong specificity in cancer and have been developed as new targets for cancer therapeutics and diagnosis.^4^ Thus, we evaluated the correlation between MDRG DNA methylation levels and gene expression as well as patient prognosis in GBM. The analysis results showed that previously uncharacterized DNA methylation sites are significantly associated with GBM patients' prognosis (Fig. 1A and Table S2, 3). Our study sheds light on identifying new biomarkers and therapeutic targets for glioma through features of MDRG methylation levels.

**Figure 1:**
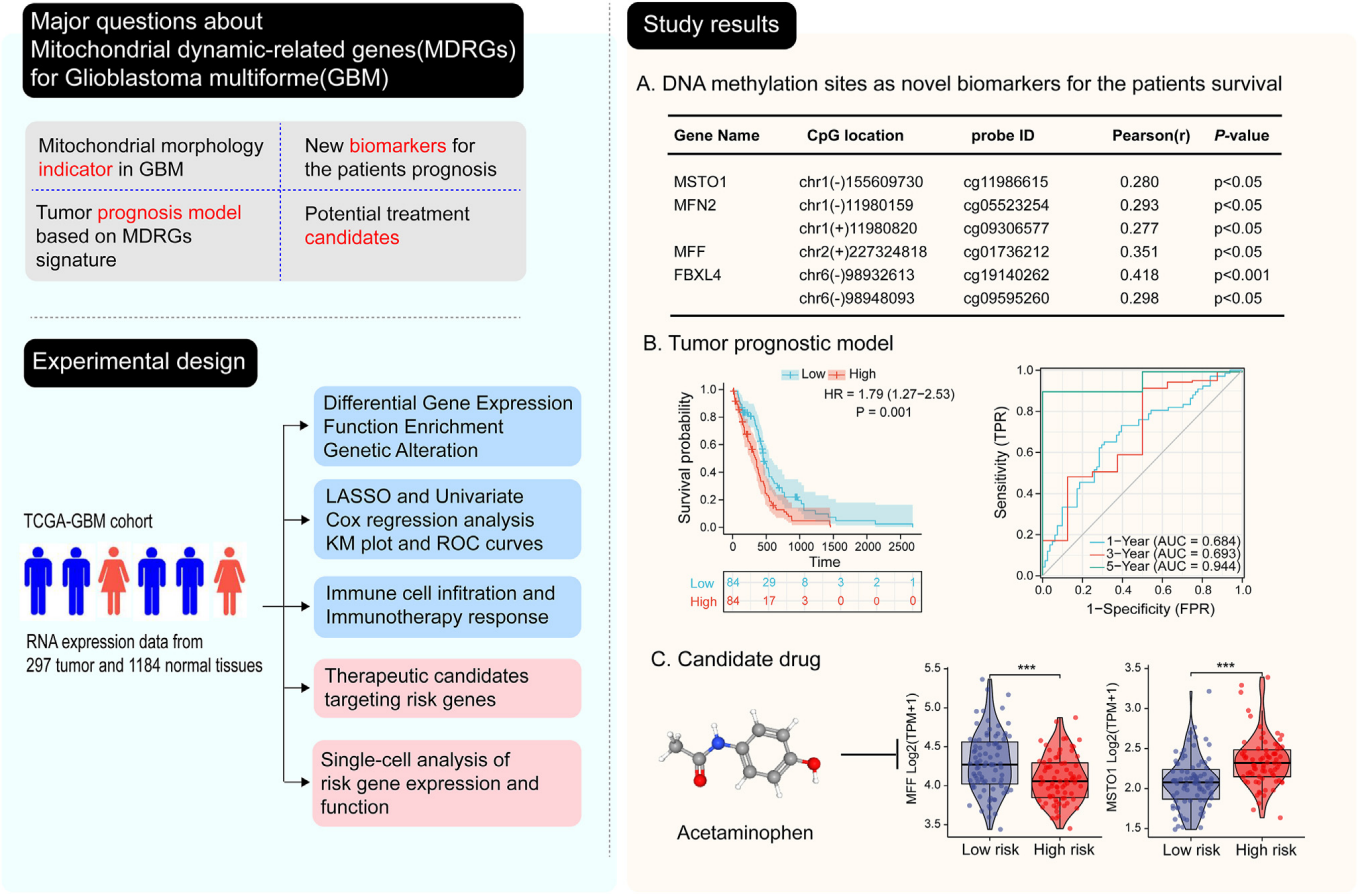
Conceptualization and visualization of the functions and prognosis values of mitochondrial dynamic-related genes (MDRGs) for glioblastoma multiforme (GBM). The following are the four major tasks to be addressed in this study: (i) whether differential expression of MDRGs serves as an indicator of mitochondrial morphology in GBM; (ii) mining new biomarkers significantly associated with GBM patients' prognosis; (iii) establishing the tumor prognostic model based on MDRGs' signature; (iv) selecting drugs with therapeutic potential targeting risk genes for GBM patients. To resolve the above issues, we analyzed transcriptomic data and corresponding clinicopathological information from the TCGA-GBM cohort to clarify the differential expression, functions, and genetic alterations of MDRGs; to develop a prognostic model based on MDRGs' signature through performing LASSO and univariate COX regression analyses; to assess the independent indexing role of MDRGs' expression and risk factors in immune cell infiltration and immunotherapeutic response; to define their oncogenic role based on single-cell analysis of risk genes' expression and functions; and to screen for possible therapeutic candidates for GBM according to the differences in expression and the available interaction evidence of risk genes. Our findings show that **(A)** DNA methylation sites associated with patient prognosis could serve as novel biomarkers, **(B)** the tumor prognosis model is accurate in predicting the survival of GBM patients, and **(C)** acetaminophen could function as a candidate drug targeting risk genes for GBM treatment. However, since both mitochondrial fusion and fission-related genes exhibited decreased expression in our analysis, we failed to phenotype mitochondrial morphology in GBM from differential expression of MDRGs. ^∗∗∗^*P* < 0.001.

Then we developed a prognostic model based on the MDRGs' signature through LASSO and univariate COX regression analyses. Firstly, we identified three MDRGs (MFF, MSTO1, and MFN1) significantly associated with patient prognosis based on the analysis of transcriptomic data of 167 GBM patients and corresponding clinicopathological information from the TCGA database using univariate and multivariate Cox regression model (Fig. S4A, C). All GBM patients were divided into high- and low-risk groups based on the medium-risk score threshold (Fig. S5A). Kaplan-Meier analysis uncovered that inferior prognosis in the high-risk group. ROC curve analysis showed that the area under the curve (AUC) of the indexing 1-, 3-, and 5-year survival was 0.684, 0.693, and 0.944, respectively (Fig. 1B). Next, we compared the prognosis value of risk factors with other clinicopathological data on GBM patients using LASSO analysis (Fig. S4B, D, H, 5H). The nomogram revealed that the MDRGs' signature was more important compared with other clinical indicators (Fig. S5B). Meanwhile, decision curve analysis results revealed that prognosis prediction is more accurate based on risk factors (Fig. S4G). The calibration curves displayed were consistent with the actual GBM patients' survival (Fig. S5C, D). The above analysis results indicated that this model based on MDRGs' signature has a good performance in predicting GBM prognosis and exhibits great accuracy in predicting prognosis.

We subsequently explored the potential application of MDRGs' expression and risk factors to estimate the immune cell infiltration level and immunotherapy response in GBM patients. The analysis showed that the expression of MDRGs was significantly associated with the infiltration level of multiple immune cells and that risk factors were more specific than MDRGs' expression in immunotherapeutic response (Fig. S6A–D). Our findings showed that the expression of MDRGs and risk factors could serve as independent indexes to estimate the level of immune infiltration in the tumor microenvironment and response to the targeted immune checkpoints.

To comprehensively understand the three risk genes' biological functions in GBM, we performed a single-cell analysis of risk genes' expression and cellular functions. The risk genes were the most abundant in malignant cells and their expression was significantly related to carcinogenesis (Fig. S7A–O). GSEA enrichment analysis showed the risk genes' functions significantly enriched in anti-tumor immune pathways (Fig. S8A–F). Targeting MDRGs has great potential for the treatment of cancers.^5^ Besides, we identified acetaminophen, which modulates risk gene expression, as a therapeutic candidate for GBM patients (Fig. 1C; Fig. S9A–F).

In summary, our study provides a new perspective for predicting GBM patients' survival and drugs targeting risk genes as novel candidate compounds for the treatment of GBM patients.

## Ethics declaration

The study was conducted according to the guidelines of the Declaration of Helsinki, and approved by the Medical Ethics Committee at the Huashan Hospital of Fudan University.

## Author contributions

Conceptualization and writing – review & editing: ZX, WH, and HW; Investigation and formal analysis: ZX; Project administration and funding acquisition: HW. All authors reviewed and approved the published version of the manuscript.

## Conflict of interests

The authors disclaim any conflict of interests.

## Funding

This work was supported by the Key R&D Program of the Science and Technology Ministry of China (No. 2021YFC2701100), the 10.13039/501100001809National Natural Science Foundation of China (No. 82150008, 81930036), and the Commission of Science and Technology of Shanghai Municipality, China (No. 20JC1418500).

## Figures and Tables

**figs1:**
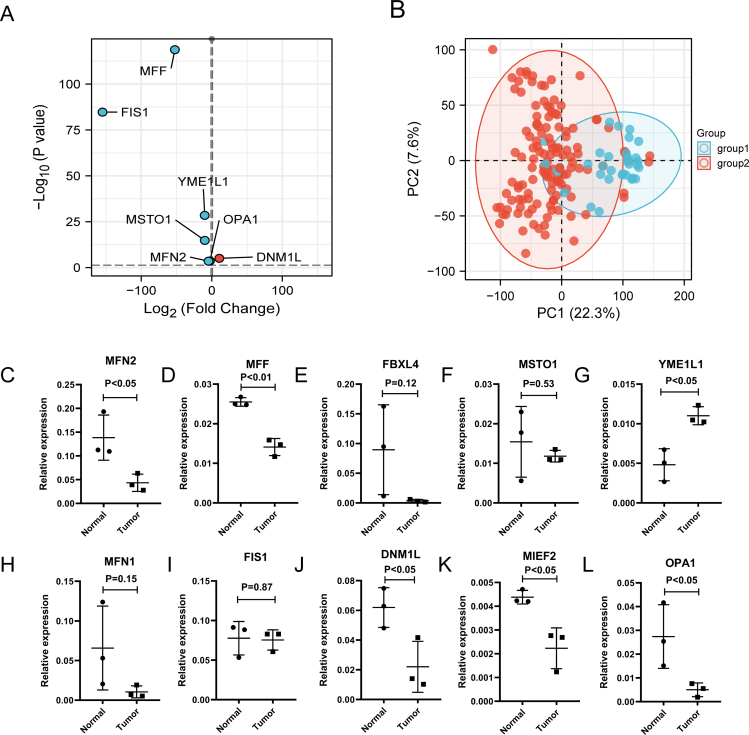


**figs2:**
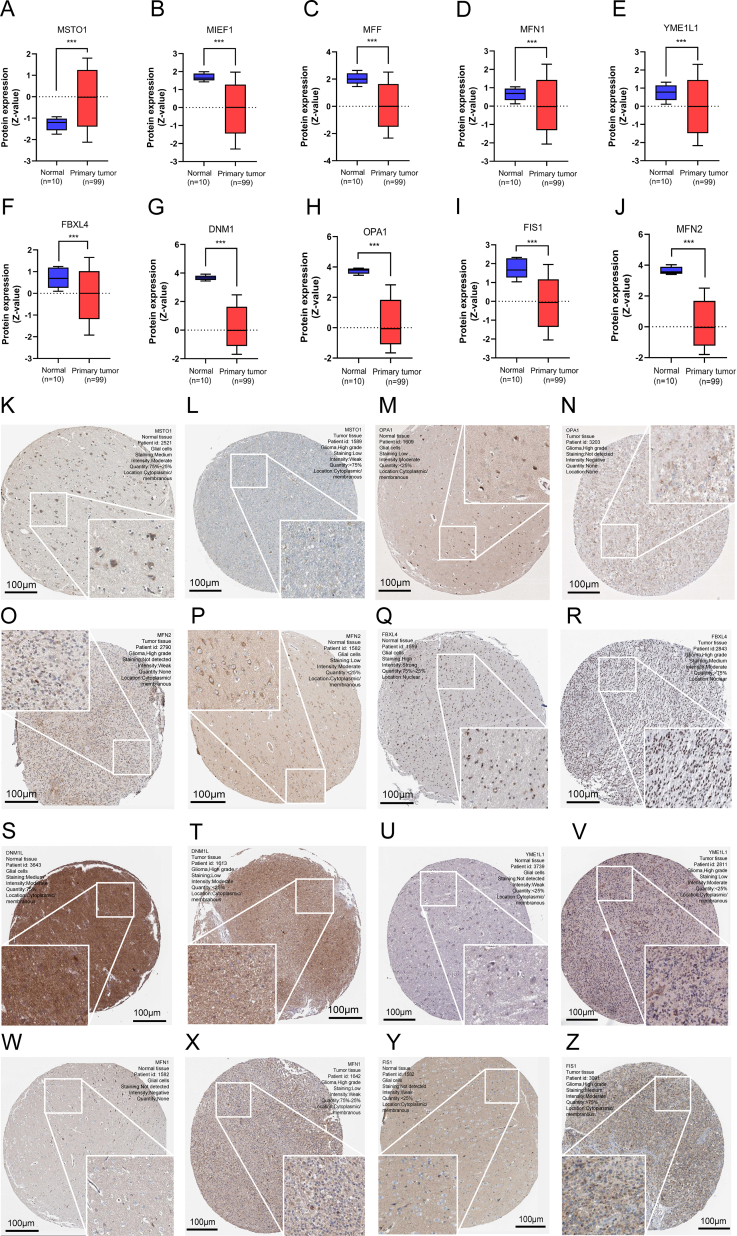


**figs3:**
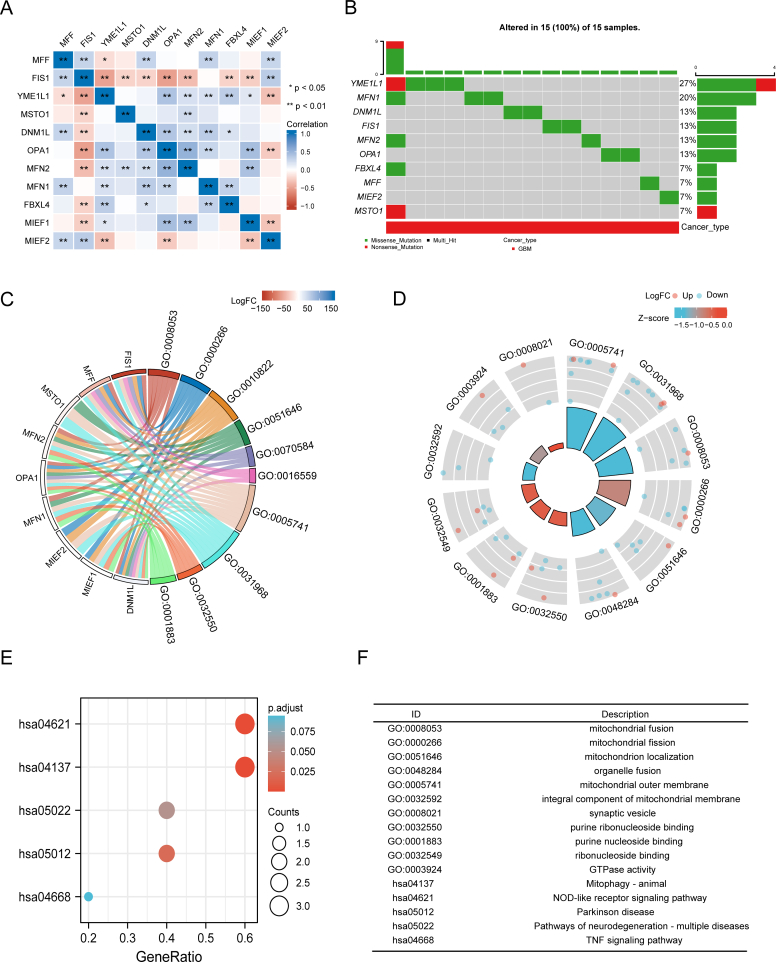


**figs4:**
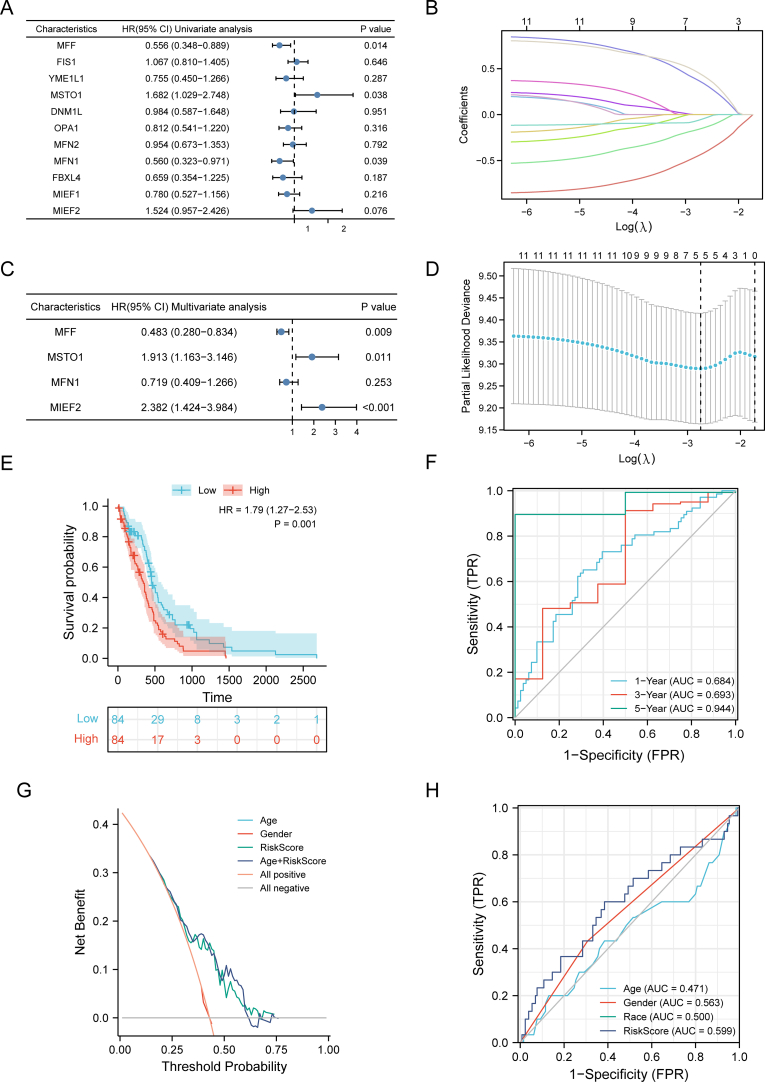


**figs5:**
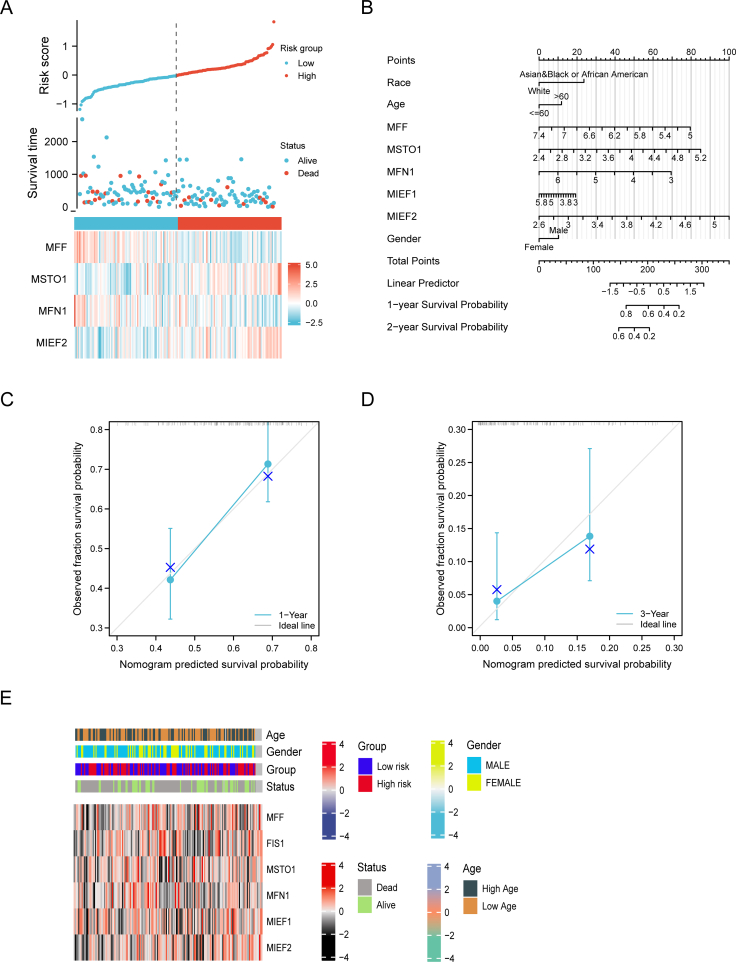


**figs6:**
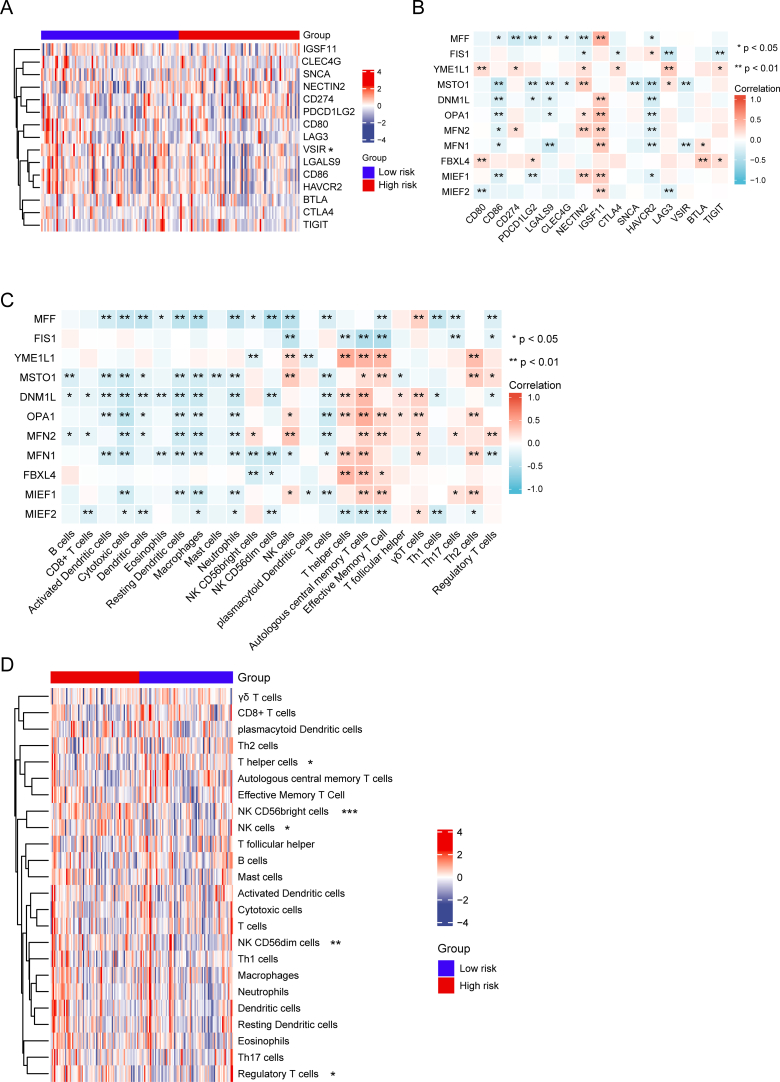


**figs7:**
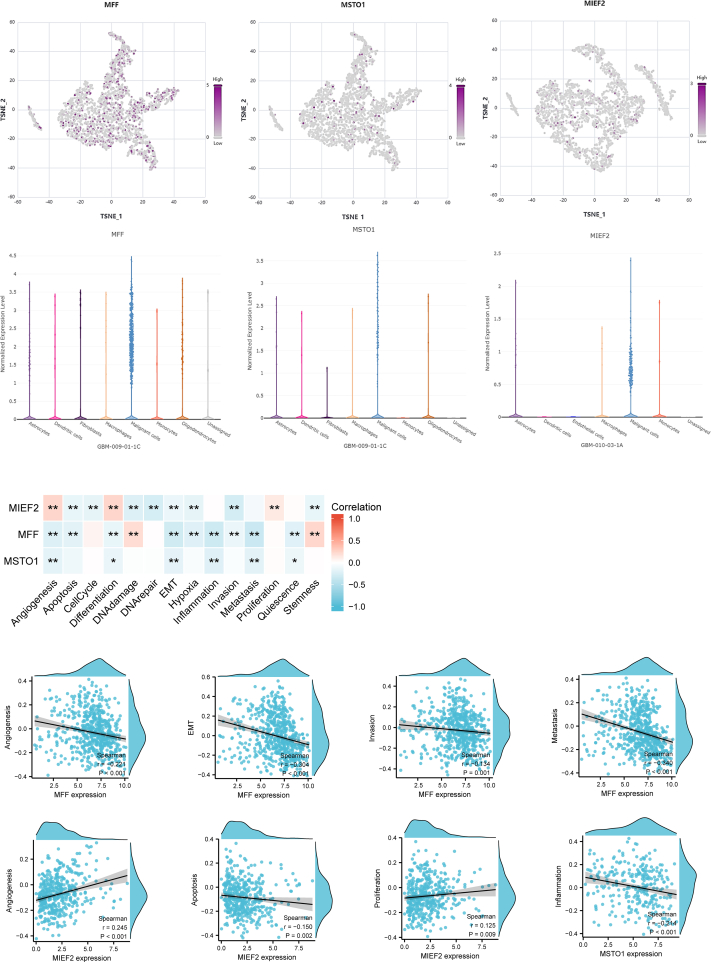


**figs8:**
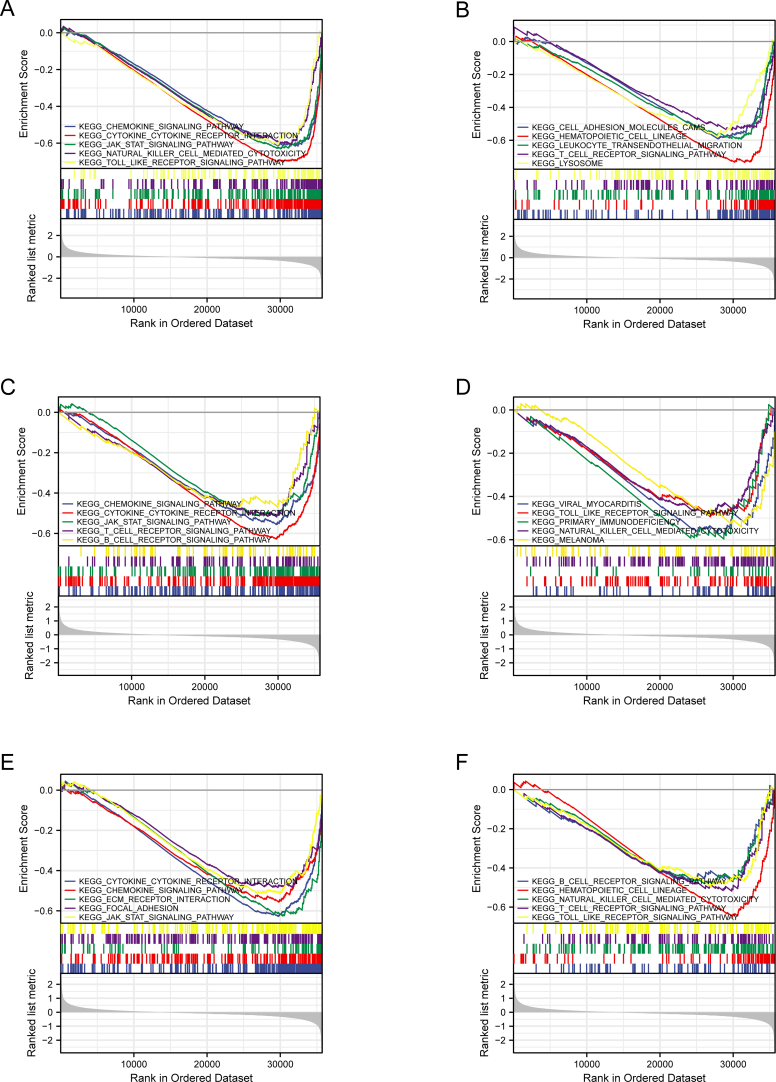


**figs9:**
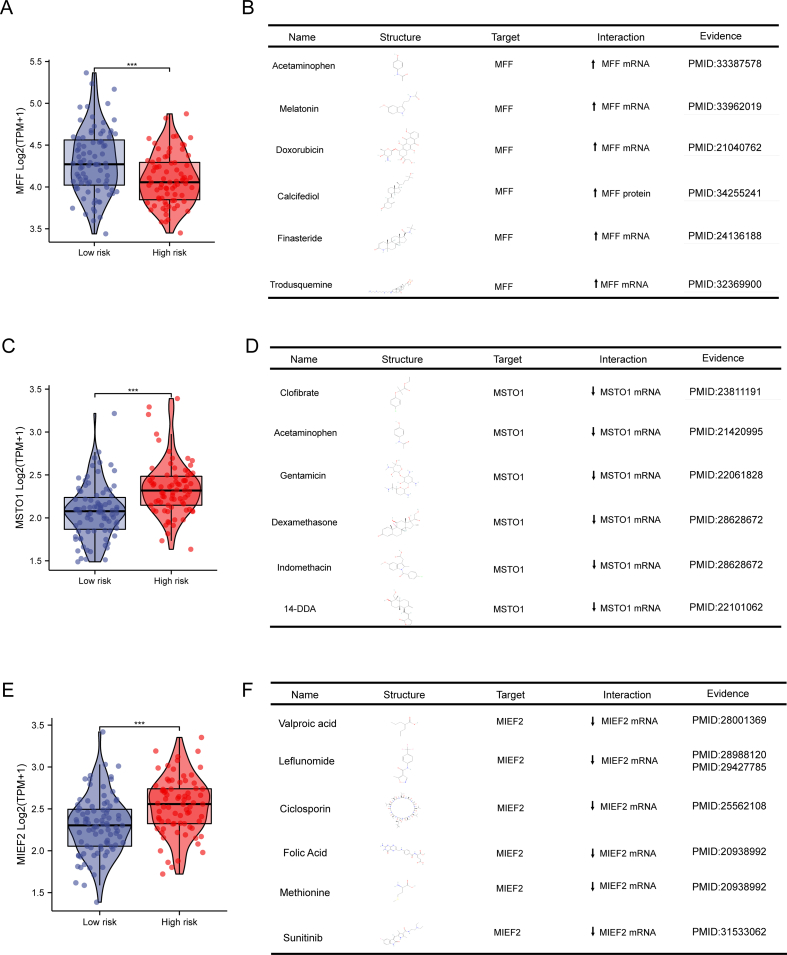


**figs10:**