# Supplementary material for: Comprehensive analysis of mitochondrial dynamic-related genes on their functions and prognostic values for glioblastoma multiforme
Source: Genes Dis. 2023 Sep 9;11(5):101084. doi: 10.1016/j.gendis.2023.101084 (PMC11177063; doi:10.1016/j.gendis.2023.101084)
Supplement: Multimedia component 1 [file mmc1.docx]

**Supplementary data**

**Materials and methods**

**Data Extraction and Preprocessing**

Transcriptomic Results of 168 patients with GBM and Corresponding

Clinicopathological information were downloaded from the Cancer Genome

Atlas (TCGA) data portal (https://portal.gdc.cance r.gov/). Normal tissue samples were collected from the GTEx databases(<https://www.gtexportal.org/home/datasets>).

GSE43289, GSE43378, GSE15824, GSE34152, GSE50161, GSE66354 and

GSE16011 were obtained from Gene Expression Omnibus

(GEO, <https://www.ncbi.nlm.nih.gov/geo/>).The protein expression level were obtained from the UALCAN portal(<http://ualcan.path.uab.edu/analysis-prot>).And the Human Protein Atlas databases ([https://www.proteinatlas.org/).](https://www.proteinatlas.org/).%20Single-cell%20data%20analysis%20was%20performed%20in%20CancerSEA(http://biocc.hrbmu.edu.cn/CancerSEA/)%20and%20CancerSEM(https://ngdc.cncb.ac.cn/cancerscem/).%20Mitochondrial) [Single-cell data analysis was](https://www.proteinatlas.org/).%20Single-cell%20data%20analysis%20was%20performed%20in%20CancerSEA(http://biocc.hrbmu.edu.cn/CancerSEA/)%20and%20CancerSEM(https://ngdc.cncb.ac.cn/cancerscem/).%20Mitochondrial)

[performed in CancerSEA(http://biocc.hrbmu.edu.cn/CancerSEA/) and](https://www.proteinatlas.org/).%20Single-cell%20data%20analysis%20was%20performed%20in%20CancerSEA(http://biocc.hrbmu.edu.cn/CancerSEA/)%20and%20CancerSEM(https://ngdc.cncb.ac.cn/cancerscem/).%20Mitochondrial)

[CancerSEM(https://ngdc.cncb.ac.cn/cancerscem/). Mitochondrial](https://www.proteinatlas.org/).%20Single-cell%20data%20analysis%20was%20performed%20in%20CancerSEA(http://biocc.hrbmu.edu.cn/CancerSEA/)%20and%20CancerSEM(https://ngdc.cncb.ac.cn/cancerscem/).%20Mitochondrial) dynamic-related

genes were identified from Yin Chieh-Fan et al.(2022). The R software limma package was carried out to analyze mRNA differential expression(DEGs). “Adjusted p < 0.01 and |log2FC| > 0.5” were defined as the threshold between tumor and normal samples.

**Enrichment pathway and function analysis**

Gene Ontology (GO), Gene Set Enrichment Analysis (GSEA) and Kyoto Encyclopedia of Genes and Genomes (KEGG) were performed with the ClusterProfiler packages. The analysis results were visualized in R software ggplot2 packages.

**Establishment and verification of the prognostic model**

The Receiver Operating Characteristic (ROC) curves and Kaplan-Meier survival analysis were carried out to predict GBM patients prognosis. Using the Lasso Cox regression algorithm and the R software glmnet package, the prognostic signature of MDRGs was constructed and the risk score based on multivariate Cox regression coefficient and each gene expression calculated. The Cox regression method was adopted to employ Uni- and multivariate analyses. A nomogram using the “RMS” package was investigated on GBM patients prognosis and calibration curves were plotted for the prediction of GBM patients 1 -, 2-year survival rate.

**Immune cell infiltration analysis**

The correlation between MDRGs and tumor-infiltrating immune cells was analyzed using TIMER algorithm. Immunomodulators were obtained from the previous literature by Thorsson V et al. (2019). The scatter plot and heatmap were employed to visualize the correlation values and P-values based on Spearman's rank test.

**Screening of potential therapeutic agents**

Based on risk genes expression in two risk groups, we identified validated drug candidates that modulate risk gene expression from the PubChem database (https://pubchem.ncbi.nlm.nih.gov/). Other Pharmacogenomic data such as the conformations of drugs in 2D was also obtained from the PubChem database.

**Reverse transcription and qPCR**

GBM patient tissues RNA was extracted using the RNA isolater reagent (Vazyme) and then was reverse transcribed using cDNA Synthesis Kit (Vazyme). qRT-PCR was carried out using SYBR Mix (Vazyme). The genes relative expression was normalized to *GAPDH* mRNA. The primers used in this study are listed in Table S4.

**Statistical analysis**

R software was performed to analyze all datas statistical significant. *p*-value<0.05 was considered as threshold value.

**Supplementary Figure Legends**

**Figure S1.** Expression analysis of MDRGs in GBM patients**.** **(A)** Volcano plot indicates the expression patterns of MDRGs in GBM. Reddots indicate high expression and bluedots indicate low expression. **(B)** PCA analysis for GBM tumor and normal groups in GEO cohort. **(C-L)** Relative expression of MDRGs in normal and tumor tissues, shown by qRT-PCR. MDRGs, Mitochondrial dynamic-related genes. Data are presented as mean ± SD. Error bars represent SD in triplicate experiments. All p-values were computed using bivariate unpaired Students' T-test; *p < 0.05, **p < 0.01, ***p < 0.001.

**Figure S2.** Total protein expression and immunohistochemical analysis of MDRGs in GBM. **(A-J)** MDRGs protein expression level between tumor and normal tissues based on the CPTAC dataset. ***p < 0.001. **(K-Z)** Immunohistochemical analysis of MDRGs in GBM from HPA database.

**Figure S3.** Genetic alterations and functional annotation of MDRGs in GBM. **(A)** Correlation analysis of MDRGs expression in GBM. **(B)** Mutation analysis of

MDRGs in TCGA cohort. **(C)** Chord plot displaying the relationship between MDRGs and GO enrichment pathway. **(D)** GO term analysis of MDRGs. The uppermost outer ring indicates the GO enrichment pathway. With the second external ring representing gene numbers in the pathway, the column heights in the internal rings depict MDRGs proportion of all genes in the pathway, and the color densities reflect the expression changes. **(E)** KEGG pathway analysis of MDRGs. **(F)** Annotation of KEGG and GO enrichment pathway.

**Figure S4.** Establishment of the risk prognostic model based on TCGA-GBM cohort MDRGs signature **(A, C)** Univ- and Multivariate Cox regression analysis between MDRGs expression and GBM patients prognosis. **(B)** LASSO regression motoring Counter-validation for fine tuned parameter options. **(D)** The LASSO coefficient profile of MDRGs signatures. **(E)** Kaplan Meier curves showing GBM patients overall survival time in two risk groups. **(F)** ROC curves for 1, 3, and 5 years display risk score with predictive effectiveness. **(G, H)** Prognostic Decision Curve and ROC curves analysis displaying risk score and other clinicopathological features.

**Figure S5.** Validation and application of prognostic model established. **(A)** The Upper section indicates the distribution of risk factors, the middle shows the survival of patients in different risk groups, and the bottom represents the expression of the four MDRGs in GBM patients. **(B)** Nomogram based on 5 prognosis-related genes and other clinical characteristics. **(C-D)** Calibration curves matched with nomogram for

predicting GBM patient survival time. **(E)** Heatmap demonstrating the association between the expression of six MDRGs and the distribution of risk scores and its clinical traits

**Figure S6.** Immune cell infiltration and immunotherapy response analysis. **(A)** Immune checkpoint gene expression in two risk group. **(B)** The expression of MDRGs is associated with immune checkpoint genes. **(C)** Relevance of MDRGs expression to immune cell infiltration. **(D)** Immune cell infiltration level in two risk group. *p<0.05, **p<0.01, ***p<0.001.

**Figure S7.** Analysis of risk genes expression and function at the single-cell level.

**(A-C)** T-SNE describes the expression profiles of risk genes in the single cells obtained from GBM samples. The dots indicate cells and the color of the dots refers to the gene expression level in that cell. The plot was downloaded from the CancerSEM database. **(D-F)** The violin plots showing the risk genes expression in different cell subtypes. The risk genes were all the most abundant in malignant cells and lower in immune cells. **(G)** Heatmap represents relevance between risk genes expression and 14 functional states. *p<0.05, **p<0.01. **(H-O)** The scatter plot indicates relationship between risk genes and oncogenic function. Statistical methods were applied with *Spearman test.*

**Figure S8.** GSEA analysis of the risk genes in GBM. **(A-F)** Top 10 enrichment pathway based on MFF, MSTO1, MIEF2 expression in GBM. The upper different color curves indicate various KEGG enrichment pathways; the middle identical color lines indicate diverse genes; the bottom denotes normalized value distribution.

**Figure S9.** Drug screening for targeting risk genes. **(A, C, E)** MFF, MSTO1, MIEF2 expression level in two risk group. **(B, D, F)** Therapeutic candidates for GBM targeting MFF, MSTO1, MIEF2. ***p<0.001

**Figure S10.** Schematic depiction of mitochondrial dynamic-related genes functions.

The biological processes involved in mitochondrial dynamics include mitochondrial fusion and fission. Those proteins related to mitochondrial fusion are MSTO1, MFN1, MFN2, OPA1, YME1L1, FBXL4. Other proteins associated with mitochondrial fission are DNM1L, FIS1, MFF, MIEF1, MIEF2. In mitochondrial fission, FIS1, MFF and MIEF1/2 localized in the outer mitochondrial membrane can recruit DNM1L that gathers into the ring to drive mitochondrial membrane division. Within the mitochondrial fusion process, MSTO1 located in the cytoplasm translocates to promote the formation of homo- or heterodimeric MFN1/2 on the outer mitochondrial membrane. While OPA1 generates two protein isoforms as a result of alternative splicing, the long isoform OPA1(L-OPA1) is catalyzed by YME1L1 to produce the short isoform OPA1(S-OPA1) that promotes mitochondrial fusion. FBXL4, which resides in the inner mitochondrial membrane space, regulates the fusion process through interactions with other mitochondrial fusion proteins.

**Supplementary Table**

**Table S1.** The profile of mitochondrial dynamics-related gene CNV (%) in GBM.

Gene symbol Het.amp. Het.del. Homo.amp. Homo.del. Total amp. Total del.

DNM1L 9.88% 8.49% 0.69% 0.17% 10.57% 8.67%

FBXL4 4.15% 23.74% 0.17% 0.35% 4.33% 24.09%

FIS1 80.76% 1.04% 1.04% 0 81.80% 1.04%

MFF 5.03% 6.76% 0 0.17% 5.03% 6.93%

MFN1 14.21% 4.85% 2.60% 0 16.81% 4.85%

MFN2 14.21% 11.26% 0.69% 0 14.90% 11.26%

MIEF1 5.89% 33.45% 0 0.17% 5.89% 33.62%

MIEF2 8.32% 11.44% 0 0.52% 8.31% 11.96%

MSTO1 17.85% 1.73% 0.87% 0 18.71% 1.73%

OPA1 13.52% 10.23% 1.04% 0.17% 14.56% 10.40%

YME1L1 2.43% 83.19% 0.17% 0 2.60% 83.19%

Het.amp.**:** Hetezygous amplification; Het.del.: Hetezygous deletion

Homo.amp.: Homozygous amplification; Homo.del.: Homozygous deletion

**Table S2.** Correlation between CpG islands methylation and their expression levels of mitochondrial dynamic related genes in GBM.

**Gene Name CpG location probe ID Pearson(r) *P-*value**

MSTO1 chr1(-)155610110 cg04706183 0.270 p<0.05

chr1(-)155613179 cg14160151 -0.269 p<0.05

FIS1 chr7(-)101245207 cg02031965 -0.326 p<0.01

chr7(-)101252524 cg00418469 -0.285 p<0.05

OPA1 chr3(+)193592743 cg04134015 -0.246 p<0.05

chr3(+)193593080 cg27222803 -0.319 p<0.05

chr3(+)193688211 cg23144852 -0.253 p<0.05

chr3(+)193696904 cg05652783 -0.266 p<0.05

MFN2 chr1(+)11978992 cg06967016 0.282 p<0.05

chr1(+)11994828 ch.1.448124R -0.332 p<0.01

YME1L1 chr10(+)27151572 cg27308434 0.410 p<0.001

chr10(-)27153889 cg11609007 -0.525 p<0.001

chr10(+)27154389 cg02094388 -0.263 p<0.05

chr10(-)27154402 cg08905758 -0.496 p<0.001

chr10(-)27154407 cg23635883 -0.413 p<0.001

chr10(+)27155025 cg08463681 0.265 p<0.05

chr10(+)27155319 cg06834274 -0.306 p<0.05

chr10(-)27158125 cg11759194 0.278 p<0.05

MIEF2 chr17(+)18260530 cg17154187 -0.282 p<0.05

MIEF1 chr22(+)39499728 cg11779973 -0.293 p<0.05

chr22(+)39502197 cg17538898 0.415 p<0.001

chr22(+)39502233 cg11878476 -0.345 p<0.01

chr22(+)39502242 cg24734643 -0.469 p<0.001

chr22(+)39502402 cg08431291 -0.533 p<0.001

chr22(+)39502411 cg02546072 -0.491 p<0.001

chr22(+)39502424 cg06456258 -0.304 p<0.05

chr22(-)39502616 cg12188538 -0.293 p<0.05

MFF chr2(-)227325542 cg07087156 -0.385 p<0.01

chr2(-)227325637 cg16944964 -0.259 p<0.05

chr2(+)227326590 cg19504888 -0.446 p<0.001

chr2(-)227327701 cg13406768 -0.505 p<0.001

DNM1L chr12(-)32678436 cg07201835 0.289 p<0.05

chr12(+)32679402 cg20180148 -0.276 p<0.05

chr12(-)32679528 cg24786579 -0.308 p<0.05

chr12(+)32679960 cg06077899 -0.450 p<0.001

chr12(+)32680034 cg01342692 -0.327 p<0.01

FBXL4 chr6(+)98947636 cg14098744 -0.349 p<0.01

**Table S3.** The correlation between methylation level of mitochondrial dynamics-related genes CpG islands and prognosis of GBM patients.

**Gene Name CpG location probe ID Pearson(r) *P-*value**

MSTO1 chr1(-)155609730 cg11986615 0.280 p<0.05

MFN2 chr1(-)11980159 cg05523254 0.293 p<0.05

chr1(+)11980820 cg09306577 0.277 p<0.05

MFF chr2(+)227324818 cg01736212 0.351 p<0.05

FBXL4 chr6(-)98932613 cg19140262 0.418 p<0.001

chr6(-)98948093 cg09595260 0.298 p<0.05

**Table S4.** The sequence of this study required primers.

NAME SEQUENCE(5’->3’)

hGAPDH-RT-F TGCACCACCAACTGCTTAGC

hGAPDH-RT-R GGCATGGACTGTGGTCATGAG

MFF-RT-F CACCACCTCGTGTACTTACGC

MFF-RT-R GTCTGCCAACTGCTCGGATTT

FIS1-RT-F GATGACATCCGTAAAGGCATCG

FIS1-RT-R AGAAGACGTAATCCCGCTGTT

YME1L1-RT-F AGGGACCTTGGATTATCTGAACT

YME1L1-RT-R TGGGATGTATGCCAATGGGAA

MSTO1-RT-F ATCCCAAGAACCCTTATCTCCA

MSTO1-RT-R GGGAGTGGTGAGGAACCTTT

DNM1L-RT-F CTGCCTCAAATCGTCGTAGTG

DNM1L-RT-R GAGGTCTCCGGGTGACAATTC

OPA1-RT-F CGACCCCAATTAAGGACATCC

OPA1-RT-R GCGAGGCTGGTAGCCATATTT

MFN2-RT-F CACATGGAGCGTTGTACCAG

MFN2-RT-R TTGAGCACCTCCTTAGCAGAC

MFN1-RT-F TGGCTAAGAAGGCGATTACTGC

MFN1-RT-R TCTCCGAGATAGCACCTCACC

FBXL4-RT-F AGAGGACGCCACCTAATTTTCA

FBXL4-RT-R GGATTTGCAGAACAAGCGAGAAT

MIEF2-RT-F CCGTGAAGCGGTTCATT

MIEF2-RT-R GTTTCTGCAGGCCCTTCTGGGG

MIEF1-RT-F GCCCAGTGCCCTAAACCCCAAG

MIEF1-RT-R CATGGCCACCAAACCCCAAC
